# Heat Conduction Model Based on the Explicit Euler Method for Non-Stationary Cases

**DOI:** 10.3390/e27100994

**Published:** 2025-09-24

**Authors:** Attila Érchegyi, Ervin Rácz

**Affiliations:** 1 Doctoral School of Applied Informatics and Applied Mathematics, Obuda University, 1034 Budapest, Hungary; 2 EVIG Engineering Co., Ltd., 1103 Budapest, Hungary; 3 Department of Natural Science, Institute of Electrophysics, Kando Kalman Faculty of Electrical Engineering, Obuda University, 1034 Budapest, Hungary

**Keywords:** transient heat conduction, explicit Euler scheme, No-Sway Threshold, finite difference method, mesh optimization, thermal diffusion, small modular reactor, Flexblue

## Abstract

This article presents an optimization of the explicit Euler method for a heat conduction model. The starting point of the paper was the analysis of the limitations of the explicit Euler scheme and the classical CFL condition in the transient domain, which pointed to the oscillation occurring in the intermediate states. To eliminate this phenomenon, we introduced the No-Sway Threshold given for the Fourier number (*K*), stricter than the CFL, which guarantees the monotonic approximation of the temperature–time evolution. Thereafter, by means of the identical inequalities derived based on the Method of Equating Coefficients, we determined the optimal values of Δt and Δx. Finally, for the construction of the variable grid spacing (M2), we applied the equation expressing the R of the identical inequality system and accordingly specified the thickness of the material elements (Δξ). As a proof-of-concept, we demonstrate the procedure on an application case with major simplifications: during an emergency shutdown of the Flexblue® SMR, the temperature of the air inside the tank instantly becomes 200 °C, while the initial temperatures of the water and the steel are 24 °C. For a 50.003 mm × 50.003 mm surface patch of the tank, we keep the leftmost and rightmost material elements of the uniform-grid (M1) and variable-grid (M2) single-line models at constant temperature; we scale the results up to the total external surface (6714.39 m^2^). In the M2 case, a larger portion of the heat power taken up from the air is expended on heating the metal, while the rise in the heat power delivered to the seawater is more moderate. At the 3000th min, the steel-wall temperature in M1 falls between 26.229 °C and 25.835 °C, whereas in M2 the temperature gradient varies between 34.648 °C and 30.041 °C, which confirms the advantage of the combination of variable grid spacing and the No-Sway Threshold.

## 1. Introduction

The numerical modeling of heat conduction plays a decisive role from nuclear power engineering to mechanical design in the prognostication of safety margins, transient processes, and material states. The classical monographs—Carslaw and Jaeger’s analytical treatment, the engineering-focused treatments by Özışık and the Hahn–Özışık volumes, and the compendia shaping everyday engineering practice, such as those by Incropera and co-authors as well as Lienhard’s textbook—established the theoretical and computational framework on which modern simulation environments are built [1,2,3,4,5].

For the numerical solution of parabolic partial differential equations, the classical stability and convergence analyses of finite difference, finite volume, and finite element procedures—above all the Courant–Friedrichs–Lewy (CFL) condition and the von Neumann (Fourier) stability analysis—have for decades provided the norms for the choice of spatial steps and time steps, particularly in the case of explicit schemes [6,7,8,9]. The historically outstanding procedures—the forward-time, central-space (FTCS) explicit Euler scheme; the backward-time, central-space (BTCS) implicit Euler scheme; and the Crank–Nicolson semi-implicit discretization—are all cornerstones of stable and accurate time marching for the heat equation, while the alternating-direction implicit (ADI) family is widely used for the efficient solution of multidimensional problems [10,11].

In the numerical solution of the heat equation, three discretization approaches are generally applied: finite differences (FDM), finite volume (FVM), and finite elements (FEM). The methodological foundations and the triad of stability–consistency–convergence have been summarized in the most renowned volumes of the field’s literature, such as Patankar’s FVM-based heat-transfer formulation, the FDM theory in the textbooks by Strikwerda and Morton–Mayers, LeVeque’s unified ODE/PDE differencing framework, Smith’s FD methodology, as well as the classical difference method stability theory by Richtmyer and Morton [7,8,9,12,13,14,15]. In time integration, the fully explicit (e.g., forward Euler) scheme is attractive due to its simplicity and massively parallelizable structure, yet it prescribes adherence to strict time step limits (CFL-like stability conditions) for the task; in contrast, implicit and semi-implicit (e.g., Crank–Nicolson, ADI) approaches allow larger time steps but require more complex computations [7,8,9,12,15,16,17,18,19,20].

In engineering practice, industrial-grade simulation platforms are widespread for thermotechnical applications—for example, notable among these are the COMSOL Multiphysics Heat Transfer Module, ANSYS Fluent, the heat transfer procedures of Abaqus/Standard, OpenFOAM, as well as the MATLAB Partial Differential Equation Toolbox, the open-source packages FEniCS, deal.II, MOOSE, Elmer, Code Aster, and GetDP/Gmsh, and the thermotechnical packages of the Modelica Standard Library—built on various numerical bases (FD/FV/FE). In each of the listed software applications, solutions are offered on different numerical bases for combined modeling of conduction–convection–radiation heat transfer [21,22,23,24,25,26,27,28,29,30]. Fitted to problems of practical size, these systems typically embody, in time and spatial discretizations that conservatively fulfill stability and accuracy considerations and in adaptive mesh and time step selection options, opportunities for users to elaborate on the engineering problem.

In the literature, for improving the accuracy of transients—especially alongside explicit schemes—we find numerous strategies: adaptive mesh refinement (AMR), locally varying grid structures, adaptive time step selection based on error estimation, and so-called super-time-stepping methods for parabolic PDEs [31,32,33,34]. The primary advantage of explicit methods is software and hardware simplicity (good parallelizability), whereas their drawback is the time step constraint tied to the CFL, which largely determines the attainable waveform fidelity and convergence rate in the transient phase.

The present research fits into this context: our aim is a restriction of the temperature-transient response of explicit Euler-based heat conduction models that ensures a physically characteristic, non-oscillatory temperature evolution even at intermediate time instants. To this end, we introduce and analyze the No-Sway Threshold condition, which is stricter than known stability criteria but constrains an interpretable parameter relationship between the spatial step and the time step. We further show that applying the condition in the transient domain systematically reduces overshoots around the target value. We derive the threshold condition relying on the Method of Equating Coefficients, generalize it to multiple spatial dimensions, and then compare it with the classical CFL condition. The validity of the theoretical results is illustrated with homogeneous and inhomogeneous one-dimensional examples—defined with various material pairings—as well as with an application case. The latter heat dissipation example is the transient heat conduction of the external pressure vessel of the Flexblue® underwater small modular reactor (SMR). Based on the open-source descriptions of the Flexblue® concept, we approximated the shell geometry and boundary conditions on the basis of our prior nuclear knowledge. In the example, we compared two grid strategies: the uniform-grid (M1) and the variable-grid (M2) simplified, single-line 3D reduction. According to the time–heat power results, the variable grid spacing (M2) “stores” a larger part of the transient heat capacitive warming in the metal and delays the heat-power delivery toward the sea. This observation was confirmed by the temperature gradient at the 3000th min in the wall of the external pressure vessel, where higher temperatures developed in the direction of heat conduction in the case of M2 than in M1. Based on the publicly available materials on Flexblue®, the model selection and boundary conditions are founded on the idealization of the oceanic heat-absorbing medium and the pressure-bearing shell approximated as cylindrical with ellipsoid end surfaces [35].

Earlier research works at our university (Obuda University) related to the topic greatly influenced our research as well—particularly that by Imre Felde and Sándor Szénási—on the high-performance (GPU-accelerated) numerical solution of heat transfer problems. Their research primarily focused on heuristic/optimization approaches to inverse heat conduction tasks (PSO, GA, FWA, etc.), which significantly affect the accuracy and runtime of transient thermotechnical identifications [36,37,38,39,40,41]. Relying on these experiences, we are developing our own target software CHeTMoS (Computational Heat Transfer Modeling Software), which currently operates with uniform grid spacing and with the limitation of differentiation parameters based on the No-Sway Threshold and into which we plan to integrate variable grid spacing.

In summary, this introduction, starting from the classical theoretical foundations of numerical heat conduction modeling and the prevalent industrial/open software ecosystem, positions our research: we introduce and examine a new threshold condition that ensures non-oscillatory behavior in the transient domain of explicit Euler-based heat conduction models, which—combined with variable grid spacing—improves physical waveform fidelity and the forecasting of short-term responses of large-scale, heterogeneous systems (for example, water–steel–air boundary conditions).

## 2. Determination of Model Parameters

### 2.1. Stability Criterion

In the world, solving problems of thermal radiation, heat convection, or heat conduction is a common engineering and physical task. These thermodynamic processes—but let us now focus exclusively on heat conduction processes—are generally time-dependent phenomena occurring in three-dimensional Euclidean space, proceeding continuously in both space and time. Fourier published his famous results on heat conduction in his book in 1822. Around the middle of the 20th century (and earlier, going back as far as Fourier’s time), scientists typically solved the problem of these processes by obtaining the mathematical solution of the descriptive partial differential equation. The framework provided by mathematical analysis ensured the continuity of the solution function. Nowadays, in today’s computer-driven world, the application of this analytical method has fallen into the background due to its complexity, and its place has been taken by computer-assisted solutions. This also means that in the solution methods, continuity has been replaced by discretization, which can be easily implemented in computer descriptive languages. As is well known, the one-dimensional, time-dependent case of heat conduction can be described by the continuous equation $\frac{\partial T(x,t)}{\partial t}=\alpha \frac{\partial T(x,t)}{\partial x^{2}}$, while in the general case, that is, in three-dimensional Euclidean space with Cartesian coordinates, it is described by $\frac{\partial T(r,t)}{\partial t}=\alpha \nabla^{2}T\left(r,t\right)$, where r=(x,y,z) are the three Cartesian spatial coordinates and *t* is time. To determine the temporal change in the temperature distribution described by the mathematical model obtained by discretizing the continuous heat conduction equation, the explicit Euler method, among others, can be applied. The fundamental prerequisite for the operation of heat conduction models applying the explicit Euler method is the fulfillment of the stability criterion, also referred to as the Courant–Friedrichs–Lewy condition (hereinafter: CFL condition) [8]. This condition is expressed in mathematical form by the inequality (1a), where α denotes the thermal diffusivity, Δt the differentiation time step or discretized time, and Δx, Δy, Δz the elementary distances in the Cartesian coordinate system. Equation (2) provides the expression of α appearing in inequality (1a), where λ is the thermal conductivity, ρ the mass density of the heat-conducting medium, and $c_{p}$ the specific heat capacity at constant pressure.

The conditions described by the inequality system (1a) clearly depend on the discretized time (Δt), the discretized spatial step sizes (Δx, Δy, Δz), and α, i.e., the thermal diffusivity as it was mentioned before. If it is assumed that the discretization step is identical in every spatial direction, inequality (1a) can be reduced to a simpler form, yielding the inequalities in (1b).

Considering the dimensionality of the investigated space, as the number of spatial dimensions increases, the constants on the left-hand sides of the inequalities in (1b) decrease (see 1/2, 1/4, 1/6). This occurs because, in order to maintain stability, heat diffusion must be considered in more directions (x,y,z), thereby increasing the denominator value in each case [42]. Mathematically, this can be expressed as follows:

(1a) $$\frac{1}{2}\geq \alpha \;\frac{\Delta t}{\Delta x^{2}};\;\frac{1}{2}\geq \alpha \left(\frac{\Delta t}{\Delta x^{2}}+\frac{\Delta t}{\Delta y^{2}}\right);\;\frac{1}{2}\geq \alpha \left(\frac{\Delta t}{\Delta x^{2}}+\frac{\Delta t}{\Delta y^{2}}+\frac{\Delta t}{\Delta z^{2}}\right)$$

(1b) $$\frac{1}{2}\geq \alpha \frac{\Delta t}{\Delta x^{2}};\;\frac{1}{4}\geq \alpha \frac{\Delta t}{\Delta x^{2}};\;\frac{1}{6}\geq \alpha \frac{\Delta t}{\Delta x^{2}}$$

(2) $$\alpha =\frac{\lambda }{\rho c_{p}}$$

For the sake of easier interpretation, it is customary to introduce the variable K to denote the right-hand sides of inequalities (1a). Thus, for one spatial dimension $K=\alpha \;\frac{\Delta t}{\Delta x^{2}}$ and for two spatial dimensions $2K=\alpha \left(\frac{\Delta t}{\Delta x^{2}}+\frac{\Delta t}{\Delta y^{2}}\right)$, while for three spatial dimensions $3K=\alpha \left(\frac{\Delta t}{\Delta x^{2}}+\frac{\Delta t}{\Delta y^{2}}+\frac{\Delta t}{\Delta z^{2}}\right)$; the stability criterion values assigned to the conduction number *K* (1/2, 1/4, 1/6) follow from the Neumann stability analysis of the discretized heat conduction equation [43,44].

During the stability analysis, it can be assumed that the so-called heat conduction Equation (3) can also be expressed in the form of a Fourier series (see Equation (4)). The meaning of the expression of the form $T_{x,n}$ appearing in (3) is as follows: in the one-dimensional case, it represents the temperature, measured in kelvin, of the elementary-sized material occupying the *x*-th spatial position in the heat-conducting medium, taken at the *n*-th time instant. Accordingly, the other terms appearing in (3) can already be easily interpreted. Here, the multiplying factor of the variable K is the temperature difference, between the central material element and its neighboring material elements.

In the complex Fourier form of this equation, i.e., in Equation (4), G is the amplification factor, *k* is the wavenumber in the usual sense, and *i* is the imaginary unit, while $e^{ikx\;\Delta x}$ is the complex Fourier basis function. If we factor out the complex Fourier basis function from both sides of Equation (4) and simplify it, the remaining complex exponential expressions can be further simplified into a cosine expression. In this way we obtain Equation (5).

Through all this, Equation (5) is a simplified form of Equation (4). According to the Neumann stability condition, if the following holds for the amplification factor of the Fourier component—|G|≤1—then the equation is stable [7] (see Equation (5)).

(3) $$T_{x,n}=T_{x,n-1}+K\left(T_{x-1,n-1}+T_{x+1,n-1}-2T_{x,n-1}\right)$$

(4) $$Ge^{ikx\Delta x}=e^{ikx\Delta x}+K\left(e^{ik(x+1)\Delta x}+e^{ik(x-1)\Delta x}-2e^{ikx\Delta x}\right)$$

(5) $$G=1+K\left(2\cos(k\Delta x)-2\right),\;\left|G\right|\leq 1$$

In the case when the inequalities according to (1a) are taken into account in the determination of the simulation parameters, then in a thermally closed system (that is, when the total energy of the thermodynamic system is constant), in stationary or quasi-stationary states, the temperature distribution of the model shows an image identical to reality. In simulation studies where intermediate, i.e., non-stationary state temperature distribution values, are also relevant, it can be proven with a small-element model, or even analytically, that the temperature change will not proceed according to a natural function. In the present case, by a natural function we mean a function where, after an initial steep ascending or descending section, the absolute value of the slope gradually decreases, and the function value slowly converges to the target value. Taking this value and the quantization of energy into account, the function value reaches the target value in a time shorter than infinity. This function form coincides with the function form that also describes the temperature equalizations due to heat conduction experienced and measurable in reality.

If the simulation parameters are close to the value determined by the above equation, then the temperatures of the model elements will describe, in mathematical terms, an oscillating curve, or in control theory terminology, an underdamped oscillatory curve, but with the amplitude of the oscillation decreasing over time.

For the execution of our analysis, let us assume a one-dimensional, closed, three-element heat conduction system, in which the material elements are positioned next to each other and realize one-dimensional heat conduction. Each material element possesses identical material properties and differs only in its temperature. The heat conduction taking place between them can be expressed using the one-dimensional discretized form of the heat conduction equation according to the following system of Equation (6). (In writing (6), the set of expressions and their meaning from Equation (3) were taken as a basis, i.e., in the system of Equation (6), the first index of the temperature T describes the relative position of the elements with respect to each other, while the second index denotes the time step. K is the dimensionless quantity derived from the heat conduction parameters and from the discretization of time and distance, as described earlier.)

(6) $$\begin{array}{cc} T_{x-1,n} & =T_{x-1,n-1}+K\left(T_{x,n-1}-T_{x-1,n-1}\right)\\ T_{x,n} & =T_{x,n-1}+K\left(T_{x-1,n-1}+T_{x+1,n-1}-2T_{x,n-1}\right)\\ T_{x+1,n} & =T_{x+1,n-1}+K\left(T_{x,n-1}-T_{x+1,n-1}\right) \end{array}$$

These three equations describe the minimal one-dimensional case. Such types of problems—when solving discretized problems—in most cases require the definition and specification of initial and boundary conditions. As boundary conditions for the problem mentioned here, the first and third equations describe the boundaries of the system, where bidirectional heat conduction cannot be examined, since with two elements only one neighboring element exists. The second (middle) equation is a discretized equation of the heat conduction process in terms of time and distance.

Hereinafter, the objective is to find the new, more accurate threshold value appearing in inequalities (1a) by investigating the extreme state in which, during the time interval Δt, the temperature difference between the material elements equalizes through heat conduction. For this purpose, as a first step, we naturally apply the stability criterion (1a) in the following way:

For the unified examination of heat conduction at Δt time intervals, let us introduce the value denoted by *L* for the temperature of the central element, while for the temperatures of its two neighbors we introduce the identically sized value denoted by *H*. As an initial condition for the problem, it can be mentioned that in a numerical example the initial temperature distribution is $T_{0}=\left\{H,L,H\right\}$. As we have seen above in inequality (1a), in the one-dimensional case the stability threshold is the dimensionless 1/2. By substituting the two different temperature values introduced (*L* and *H*) and the CFL condition into the system of Equation (6), we obtain the system of Equation (7). The two boundary conditions of the heat conduction model are defined by the first and last equations of the system of Equation (6), which—assuming heat conduction only toward the central element—describe a one-dimensional, time-dependent, closed heat conduction system. The second (middle) equation of the system of Equation (6) is a discretized version of the continuous equation presented above, and the other two equations are related to the boundaries. System of Equation (7) has three terms, but the temperature values referring to the two boundary material elements coincide; therefore, for these values (H+L)/2 is the result.

(7) $$\begin{array}{cc} H+\frac{1}{2}\left(L-H\right) & =\frac{H+L}{2}\\ L+\frac{1}{2}\left(H-L+H-L\right) & =H \end{array}$$

The system of Equation (7) provides the temperature values of the heat conduction transport process at the first time step (Δt=1). As described above, the temperatures of the two boundary elements will be the arithmetic mean of *H* and *L*, while the temperature of the central element will be *H*, that is, we can state that the temperature of the central material element coincides with the initial temperature of the boundary material elements. Since the temperature of each examined material element remains within the closed interval determined by the temperature values *H* and *L*, and by repeated substitution into the system of Equation (6), the temperature difference between the elements always decreases; it appears to be confirmed that adherence to the CFL condition results in a stable computation (the final state will be established only with oscillations around the final state, or, using control engineering terminology, with damped oscillations around the final state).

The heat conduction transport process in the thermodynamic system strives to equalize the temperature difference between the material elements. In other words, this means that the internal energy of the individual material elements becomes identical. In the examined example, each material element is identical, differing only in its temperature. Accordingly, in the above case, for three material elements the stationary-state temperature can be determined as the arithmetic mean of the temperatures of the three material elements. This is described by Equation (8).

(8) $$\frac{2H+L}{3}$$

**Figure 1:**
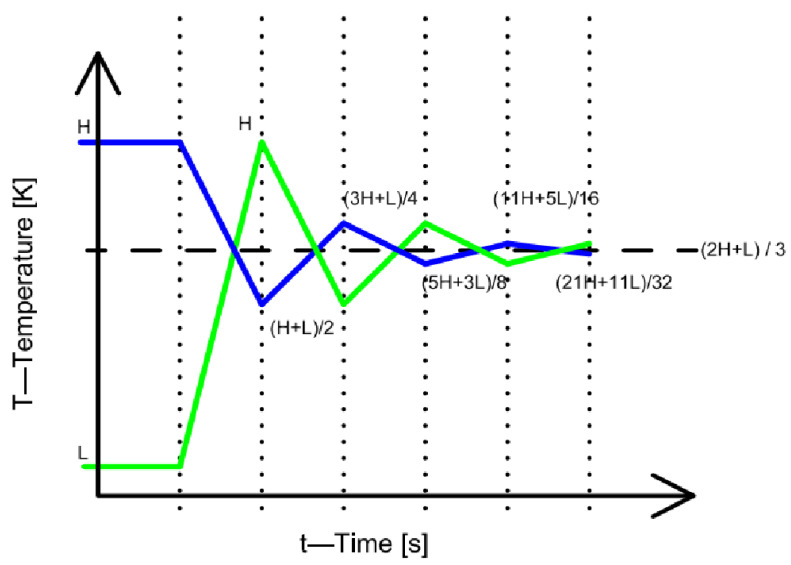
Temperature variation in the one-dimensional thermodynamic system containing three material elements during heat conduction as a function of time after five time iteration steps. The temperature marked in green corresponds to the central element, while the temperature marked in blue corresponds to its each neighboring elements.

Based on the above, the results were recursively substituted back into the system of Equation (6). This back-substitution was carried out over five iterations. The results thus obtained are presented in Figure 1. In Figure 1, the green broken line indicates the evolution in time of the temperature of the central material element over five consecutive time steps (in other words, five iterations), while the blue broken line shows the evolution of the temperature of the material elements neighboring the central material element over the same five consecutive time steps (the same five iterations). In the applied coordinate system, the vertical axis represents the temperature measured in kelvin, while the horizontal axis represents the time axis. The vertical dotted straight lines refer to the positions of the time iterations. The figure is schematic but drawn to scale. In Figure 1, the expected target temperature specified in the system of Equation (8) (which the system tends to reach with the passage of time) is indicated by a horizontal, black dashed line.

In Figure 1, the periodic temperature variations with decreasing amplitude can be observed. In each period, the slope of the plotted function changes sign. The above confirms the statement that the system will reach the stationary state, which—as shown above—in this case can be obtained as the arithmetic mean of the temperatures of the three material elements. It can also be established from the labels attached to the broken lines in the figure that the local extrema of the periodically varying function are fractional values, where the denominator is consecutively a power of two, while the denominator of the target value is three. The calculated results refer to quantized time instants, that is, where the value of Δt is always some positive integer. Regarding the arithmetic sequences formed from the results thus obtained, it can be stated that since both sequences contain terms whose denominators are even integer multiples of a prime number (powers of two with even exponents), which differ from the prime number found in the denominator of the target value (three, as a prime number), no Δt time instant exists at which the temperature of the material elements would equal the target temperature value, that is, the arithmetic mean of the initial temperatures of the material elements.

In addition to the above mathematical reasoning, it is important to note that in reality the quantization of energy, and in computer simulations the limitation of number representation, i.e., the type of variable applied, impose a lower bound on the magnitude of the temperature change. In connection with the given example, the proof of the constancy of the system’s total energy can be found in Appendix A.

### 2.2. No-Sway Threshold

There are, however, cases when it is important that the model provides good results not only in the stationary state but also in the intermediate states of the finite difference model. Such a case may be, for example, the analysis of the heating system of a building, where the study of the control of tempering may be influenced by a temperature variation with a characteristic curve disturbed by a Δt-periodic disturbance. Similarly, in a thermomechanical simulation, much greater mechanical load may result from the steeper ascending and descending slopes of temperature changes and from the periodic reversal of these slopes.

Taking all this into account, it may be necessary to apply a model that reflects a temperature distribution close to reality also in transient states. We assert that it is possible to set for the simulation such a differentiation time and step size for which the value characterizing the stability of the model, as defined by the system of inequalities (1a) and inequality (2), is smaller than the stability criterion, and thus the heat conduction proceeds according to a natural function. We refer to this new stability-characterizing value of the model as the **No-Sway Threshold**. Its value depends on the number of dimensions and is thus determined according to the three inequalities given in (9).

(9) $$\begin{array}{cc} \frac{L+2H}{3} & \geq L+K\left(2H-2L\right)\;\frac{1}{3}\geq K\\ \frac{L+4H}{5} & \geq L+K\left(4H-4L\right)\;\frac{1}{5}\geq K\\ \frac{L+6H}{7} & \geq L+K\left(6H-6L\right)\;\frac{1}{7}\geq K \end{array}$$

In the inequalities in (9), we assumed the case in which, in the one-dimensional model, the temperature of the central examined material element is *L*, while the temperature of both its neighboring material elements is *H*. Similar to the example presented by the system of Equation (6), the material elements are identical in all their physical parameters except for their temperatures. Into the second equation of the system of Equation (6), that is, into the right-hand side of the heat conduction equation of the central material element, the temperatures were substituted. Assuming that the heat equalization between the material elements takes place within a Δt time interval, the arithmetic mean of the temperatures of the material elements, that is, the target value, was substituted into the left-hand side of the equation.

If we assume that the entire system does not reach the target value within a Δt step time interval but within a longer time interval than Δt, then, upon substitution into the second equation of the system of Equation (6), the original equality sign transforms into an inequality sign. Simplifying the inequality, we obtain $K\leq \frac{1}{3}$.

By analogy with the one-dimensional case, the first inequality of (9) can also be extended to two and three spatial dimensions, taking into account the increase in the number of neighbors of the central material element when increasing the number of spatial dimensions. The number of neighbors of the central material element is four in the case of two spatial dimensions and six in the case of three spatial dimensions. In these cases, assuming the temperature *H* for all elements neighboring the central material element, the second and third inequalities of (9) result.

Comparing all this with the values of the CFL condition given in (1a) and (1b), when applying the No-Sway Threshold, in each spatial dimension the denominator of the fraction is exactly one greater. Since the stability criterion states that the model is stable if its parameters take on numerical values equal to or smaller than the value given by the criterion, the No-Sway Threshold values determined by us are smaller than the numerical values of the stability criterion. All this therefore means that with the condition determined by us, our model is stable. In the intermediate states calculated by the model, the temperature values show no oscillations; in other words, it can be stated that the temperature values converge more accurately throughout toward the real temperature values.

Every heat conduction system strives for a stable (equilibrium) state; in the case of closed systems, this occurs when all elements reach an identical temperature. The members of the system of equalities (9) were determined on the basis of the principle of striving for a stable state. Through the numerical example of the discretized form of the heat conduction equation, the No-Sway Threshold values were determined for one-, two-, and three-dimensional cases. The two sides of the system of equalities become equal in the case when the entire process of heat conduction takes place during the specified Δt time. If the model is composed of elements of homogeneous material and identical size, which differ only in their initial temperature, then the left-hand side of the system of equalities is given by the arithmetic mean of the temperatures of the elements. In the one-dimensional case, this arithmetic mean is formed from three temperature values, in the two-dimensional case from five, and in the three-dimensional case from seven temperature values. On the right-hand side of the system of equalities, the initial temperature of the central material element appears, along with the sum of the initial temperature differences, multiplied by the heat conduction number *K*. The system of equalities can be rearranged to express *K*. As a result, for the system of equalities (9) we obtained the values $\frac{1}{3}$, $\frac{1}{5}$, and $\frac{1}{7}$ for the No-Sway Threshold, depending on the number of spatial dimensions.

Regarding the general operation of heat conduction and the behavior of the case of the discretized heat conduction equation using the No-Sway Threshold, the following points are important to emphasize:

(a) Based on the second law of thermodynamics, or more narrowly the principle of minimum energy, in the case of a closed system, the system and all its elements tend toward the lowest-energy state. In the previously defined heat conduction system, this occurs when all elements of the system reach identical temperature. Our derivation assumes that the system reaches its constant-temperature state within Δt time. It can be established that, for the time step following the attainment of the stable state, the value of the bracketed term on the right-hand side of both the heat conduction equation and the boundary conditions described in the system of Equation (6) becomes zero, since there is no temperature difference between the elements of the system. When the No-Sway Threshold is observed, it is guaranteed that, at least in the case of a closed system of homogeneous material quality, after reaching the stable state, there does not exist any (future) time instant $t_{n}$ at which the temperature of the system elements would change.
(b) If the system is not closed, i.e., the total internal energy of the system changes or can change over time during the process, then the system has no true stable (equilibrium) state, but rather the quasi-stable state is always determined by the total (resultant) system energy corresponding to the given moment of time. When using the No-Sway Threshold, if, for example, the material elements located at the model boundary do not coincide with the actual physical system boundaries, then the temperature change in these affected (i.e., boundary) material elements is determined not only by the heat conduction differential equation. Since the defined heat conduction number *K* depends only on the material properties of the model, the differentiation distance, and the differentiation time, its value limits the temperature change even in the case of an extreme change in the temperature difference. Thus, even in the case of a system that is not closed and not of homogeneous material quality, it is still true that when the No-Sway Threshold is applied, the time evolution of heat conduction does not show oscillatory behavior, provided that the external influence is also not of an oscillatory character.
(c) If the material properties of the system elements change as a function of temperature, then under the initial conditions—that is, when calculations are carried out with the differentiation time and differentiation distance determined by the system of equalities (9)—it may occur that the elements of the system exhibit oscillatory temperature change. It is, however, important that at those time instants where the temperature change shows oscillatory character, the value of the heat conduction number *K* will exceed the value of the No-Sway Threshold. In practical applications where the temporal change in physical parameters (density, heat capacity, thermal conductivity) is permitted, one must first determine, within the expected temperature range, such a permissible state considered extreme, in which the value of *K* reaches its attainable absolute maximum within the examined time interval, and from that point on it determines the differentiation distance and differentiation time in the model.

In our further derivations, we also determine the ideal value of *K*. We can speak of an ideal value of the heat conduction number when the function value determined by the discretized heat conduction equation always monotonically approaches the instantaneous stable state that can be derived from the current resultant system energy of the system.

## 3. Comparison of Model Parameter Criteria

### 3.1. Examination of a Numerical Example—One-Dimensional Case

In the following, with the help of a few numerical examples, we present the practical application of the No-Sway Threshold introduced above. In the one-dimensional cases to be presented, by selecting the differentiation time (Δt) and differentiation distance (Δx) already introduced in theory, we examine models with stability characteristics (*K*, dimensionless) below and above the threshold. By comparing these, we prove that the No-Sway Threshold defined by us is indispensable for the accurate calculation of temperature values in the non-stationary states of the simulation.

In our first numerical example, the selected material is silver, for which we assume one-dimensional ideal heat conduction and whose material properties are given in Table 1. Similarly to the general case discussed in Section 2, we examine only three adjacent material elements as described in the theoretical part, such that the temperature of the central material element is lower than that of its two neighboring elements. Let the initial temperature of the central material element be *L* = 18 °C, and let us consider *H* = 24 °C as the initial temperature of its two neighboring material elements. The differentiation time is set as Δt = 1 s in each model run. The only difference between the two simulation runs is the differentiation distance.

Namely, in the first model run, the differentiation distance is set as Δx = 0.024 m. With this and the other parameter values, the stability characteristic of the model results in *K* = 0.336 (dimensionless), which is above the No-Sway Threshold numerical value defined by us (*K* = 1/3) (0.336 > 1/3). In the second model run, the differentiation distance is Δx = 0.0241 m, in which case the stability characteristic of the model results in *K* = 0.333, and this value is smaller than the No-Sway Threshold value (again *K* = 1/3). Based on the preliminary calculations of the resulting *K* stability characteristic values of the model, even before calculating the temporal evolution of the element temperatures—based on the theory presented—it could already be inferred that in the first case the characteristic curve of the temperature changes would show an overshoot with respect to the target temperature.

**Figure 2:**
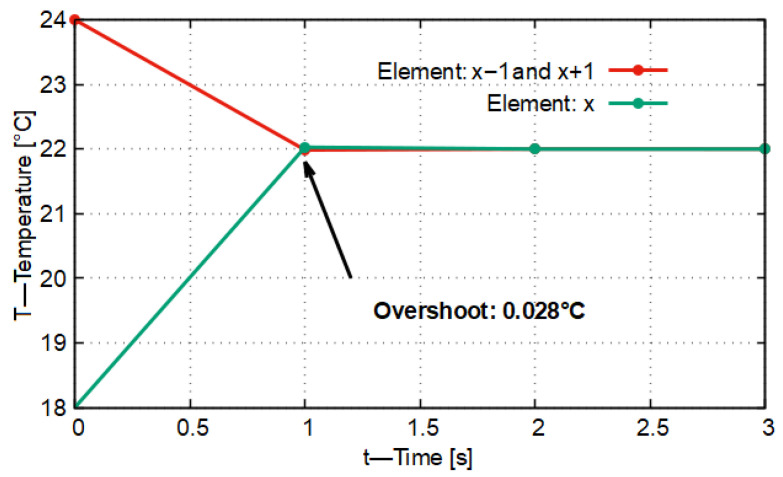
The behavior of a three-element silver material 1D heat conduction model in the first time steps when *K* is over the No-Sway Threshold. The temperature marked in green corresponds to the central element, while the temperature marked in red corresponds to its each neighboring elements.

In Figure 2 the result of the first model run can be seen. In this case it can be observed that, by approaching the predetermined No-Sway Threshold value of *K* = 0.336 from above by 0.003, during the temporal evolution of heat conduction the temperature of the central material element becomes higher by 0.028 °C than the target temperature value of 22 °C.

**Figure 3:**
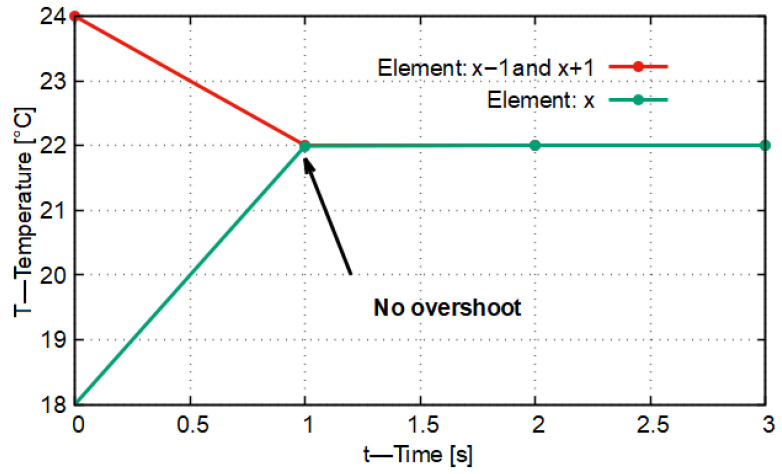
The behavior of a three-element silver material 1D heat conduction model in the first time steps when *K* is under the No-Sway Threshold. The temperature marked in green corresponds to the central element, while the temperature marked in red corresponds to its each neighboring elements.

In Figure 3 it is shown that during the first time step (Δt = 1 s) none of the material elements reached the target temperature value, and no inflection points appeared in the curves, that is, the slope did not change sign. In this case the stability characteristic of the model (*K* = 0.336) was in the vicinity of the No-Sway Threshold. The deviation of the stability characteristic from the No-Sway Threshold is ΔK = 0.003. From this it follows that all this caused only a transient deviation of 0.028 °C compared to the expected target temperature value.

If we compare this with what is given in the original CFL condition (where *K* = 0.5), then, running the model for this case, we obtain the result that, with a predetermined stability characteristic of *K* = 0.498, the temperature of the central material element exceeds the target temperature by 1.978 °C within the time step of Δt = 1 s. On the basis of all this, it can be stated that the application of the No-Sway Threshold in the case of the one-dimensional heat conduction model, assuming a homogeneous material environment, demonstrably provides more accurate results for the non-stationary time instants than the heat conduction model, which takes into account exclusively the stability criterion but is otherwise identical in all other respects.

### 3.2. Determination of the Parameters of the General Heat Conduction Model

It is also worthwhile to examine the operation of the No-Sway Threshold in the case of an inhomogeneous system. In our case, by an inhomogeneous system we mean a system in which the system is not composed solely of one type of identical material but is formed by at least two media with different material properties. In this example, among the three examined material elements, let the central element be simple rock salt, while the two boundary material elements are silver. (Silver was chosen as the boundary element because silver is known to be one of the chemical elements with the best heat conduction properties.) The physical parameters of rock salt used differ significantly from those of silver, which thus highlights more clearly the effects observed in inhomogeneity. The parameter values applied in the calculations are listed in Table 1.

The resultant heat conduction of two elements with different thermal conductivities can be expressed according to Equation (10), where $\lambda_{1}$ and $\lambda_{2}$ are, in this order, the thermal conductivity coefficients of one and the other material element.

(10) $$\lambda_{e}=\frac{2\;\lambda_{1}\lambda_{2}}{\lambda_{1}+\lambda_{2}}$$

In the heat conduction equation written for this case, the other parameters of the thermal diffusivity coefficient (*K*)—the heat capacity and the density—were substituted by the physical parameters of the examined material element. See Equation (11) written for the one-, two-, and three-dimensional cases.

(11) $$\begin{array}{cc} K_{1D} & =\frac{\lambda }{\rho c_{p}}\cdot \frac{\Delta t}{\Delta x^{2}}\\ 2K_{2D} & =\frac{\lambda }{\rho c_{p}}\left(\frac{\Delta t}{\Delta x^{2}}+\frac{\Delta t}{\Delta y^{2}}\right)\\ 3K_{3D} & =\frac{\lambda }{\rho c_{p}}\left(\frac{\Delta t}{\Delta x^{2}}+\frac{\Delta t}{\Delta y^{2}}+\frac{\Delta t}{\Delta z^{2}}\right) \end{array}$$

In the examined example, the initial temperature of the two boundary silver material elements is 24 °C, while the temperature of the central rock salt element is 18 °C. The determination of the conduction number (*K*) close to the No-Sway Threshold in this case is more complicated and differs from the calculation formula already presented. With the calculation given in Equation (11), under the parameters of step size 0.010 m and differentiation time 8.500 s, the conduction number of the central material element reaches the No-Sway Threshold.

When calculating the temporal variation in the temperature values, it can be observed that the target temperature is reached within (3·Δt) time, whereas the target temperature should be reached within (1·Δt) time for the temperature of the central material element at the vicinity of the No-Sway Threshold. This phenomenon shows that either the differentiation time can be further increased or the differentiation distance can be decreased.

For the two boundary material elements, under the parameters of step size 0.010 m and differentiation time 9.150 s, the conduction number reaches the No-Sway Threshold. When calculating the temporal temperature variations, it can be observed that the system is indeed stable, but the temperature values reach the target value in an oscillatory manner. Based on the results, it can be stated that the optimal value of the differentiation time must lie somewhere between 8.500 s and 9.150 s. For its precise determination, a generalization of Equation (11) is required, that is, taking into account the differing physical parameters of the adjacent elements in the determination of the conduction number, in the following manner (see system of Equation (12)):

(12) $$\begin{array}{cc} T_{A,n} & =T_{A,n-1}+A_{A}\lambda_{A0}\;\left(T_{0,n-1}-T_{A,n-1}\right)\\ T_{B,n} & =T_{B,n-1}+A_{B}\lambda_{B0}\;\left(T_{0,n-1}-T_{B,n-1}\right)\\ T_{0,n} & =T_{0,n-1}+A_{0}\left[\lambda_{A0}\;\left(T_{A,n-1}-T_{0,n-1}\right)+\lambda_{B0}\;\left(T_{B,n-1}-T_{0,n-1}\right)\right] \end{array}$$

The system of Equation (12) expresses a general three-element one-dimensional heat conduction model. In this case, each material element possesses different material or physical properties. For better simplification, the thermal conduction number K can be decomposed into the thermal conductivity coefficient λ between two neighboring elements and the expression “*A*.” Here, “*A*” represents the differentiation time and the differentiation distance, as well as the heat capacity and the mass density, in accordance with Equation (12). In the following derivation, the goal is to determine a general formula of the thermal conduction number *K*.

**Figure 4:**
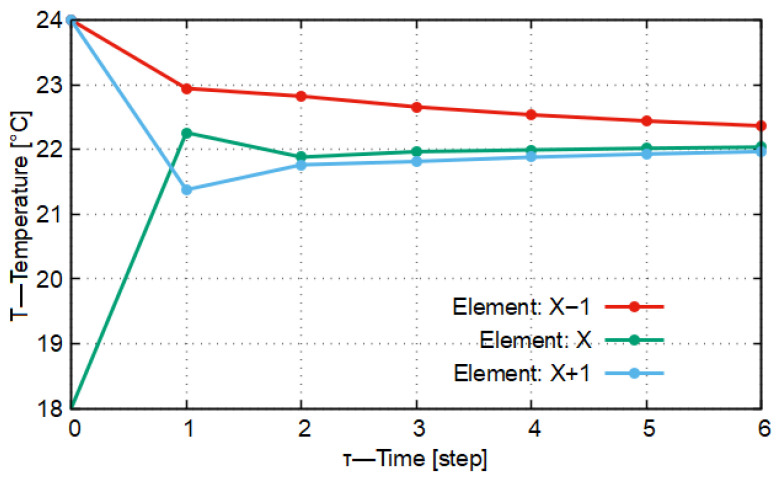
The behavior of a three-element (silver, rock salt, HDPE material) general-case 1D heat conduction model in the first time steps when *K* is over the No-Sway Threshold. The temperature marked in green corresponds to the central element (rock salt), while the temperature marked in red (silver) and blue (HDPE) corresponds to its each neighboring elements.

**Figure 5:**
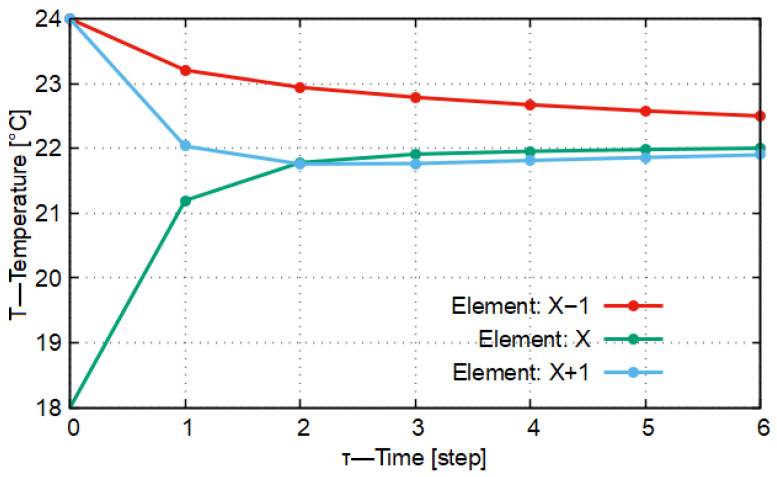
The behavior of a three-element (silver, rock salt, HDPE material) general-case 1D heat conduction model in the first time steps when *K* is under the No-Sway Threshold. The temperature marked in green corresponds to the central element (rock salt), while the temperature marked in red (silver) and blue (HDPE) corresponds to its each neighboring elements.

Let us assume that our case is a one-dimensional heat conduction model with three material elements. Each material element is of different material quality (here, silver, rock salt, and High-Density Polyethylene = HDPE). By this definition, their heat conduction, heat capacity, and density are also different. Based on the system of Equation (12), the heat conduction occurring among them can be written. In Figure 4 and Figure 5, with the differentiation length and time parameters Δx = 0.010 m, Δt = 14 s, and Δt = 10.5 s, the temperatures of the elements were calculated.

In Figure 4, it is shown that the temperature of the material element with identifier X−1 decreases strictly monotonically to the target temperature (curve marked in red in Figure 4). In the monotonicity of the function, there is an anomaly between time steps *t* = 1 and *t* = 3. The function curve of the temperature variation in the central element with identifier *X* (marked in green) is not monotonic. Between the mentioned time steps *t* = 1 and *t* = 3, the temperature reaches a local maximum, after which the temperatures decrease; then, the direction of monotonicity changes, that is, the function curve begins to increase strictly monotonically. A similar change characterizes the material element with identifier X+1 (blue curve in Figure 4), only there the temperature begins to decrease earlier. It can be stated that, with these Δt and Δx differentiation parameter settings, the system is indeed stable, but it does not meet the qualitative mathematical requirements imposed by the No-Sway Threshold in connection with temperature variations, in other words, the requirement that the function of temperature variation should be monotonic in all cases.

In Figure 5, the function curve of temperature variation has no inflection point. For the two boundary elements (X−1 (red), X+1 (blue)), the threshold value of the conduction number (*K*) calculated remains below 0.333 in this case. For the central element, two different values result for *K*, depending on which neighboring material element’s heat conduction is taken into account. In one case, the value of *K* is 0.119, while in the other case it is 0.413. In the general case, based on the calculated values, it cannot be established whether the model oscillates around the target value or not. Based on Figure 5, it can be stated that in the temperature variations of the elements with identifiers *X* and X+1 there is no longer oscillatory-type behavior. Accordingly, in summary it can be stated that the threshold values defined in the system of Equation (9) can also be used in the general case; however, they do not provide the optimal differentiation time and differentiation distance.

For the method of calculating the optimal differentiation time and distance, a function analysis is required. In the case of a thermally closed system, if there is no heat source within the system, then the monotonicity of the function of temperature variations will be determined by the first two sections of the function curves. As a first step, the temperature values corresponding to the time steps *t* = 0, *t* = 1, and *t* = 2 must be determined. The initial temperatures are known. Into the system of Equation (12), all the known temperatures can be substituted. For the sake of abbreviation of the equations, the element at position X−1 was denoted by A and the element at position X+1 by B, while the central element was denoted by the subscript 0.

(13) $$\begin{array}{c} T_{A}\left(0\right),\;T_{B}\left(0\right),\;T_{0}\left(0\right) \end{array}$$

(14) $$\begin{array}{cc} T_{A}\left(1\right) & =\left[1-A_{A}\lambda_{A0}\right]\;T_{A}\left(0\right)+A_{A}\lambda_{A0}T_{0}\left(0\right)\\ T_{B}\left(1\right) & =\left[1-A_{B}\lambda_{B0}\right]\;T_{B}\left(0\right)+A_{B}\lambda_{B0}T_{0}\left(0\right)\\ T_{0}\left(1\right) & =\left[1-A_{0}\lambda_{A0}-A_{0}\lambda_{B0}\right]\;T_{0}\left(0\right)+A_{0}\lambda_{A0}T_{A}\left(0\right)+A_{0}\lambda_{B0}T_{B}\left(0\right) \end{array}$$

(15) $$\begin{array}{cc} T_{A}\left(2\right) & =T_{A}\left(1\right)+A_{A}\lambda_{A0}\left(T_{0}\left(1\right)-T_{A}\left(1\right)\right)\\ T_{B}\left(2\right) & =T_{B}\left(1\right)+A_{B}\lambda_{B0}\left(T_{0}\left(1\right)-T_{B}\left(1\right)\right)\\ T_{0}\left(2\right) & =T_{0}\left(1\right)+A_{0}\left[\lambda_{A0}\left(T_{A}\left(1\right)-T_{0}\left(1\right)\right)+\lambda_{B0}\left(T_{B}\left(1\right)-T_{0}\left(1\right)\right)\right] \end{array}$$

(16) $$\begin{array}{cc} T_{A}\left(2\right) & =T_{0}\left(0\right)\left[2A_{A}\lambda_{A0}-A_{A}\lambda_{A0}A_{0}\lambda_{A0}-A_{A}\lambda_{A0}A_{0}\lambda_{B0}-A_{A}\lambda_{A0}A_{A}\lambda_{A0}\right]\\ \; & \;+T_{A}\left(0\right)\left[1-2A_{A}\lambda_{A0}+A_{A}\lambda_{A0}A_{0}\lambda_{A0}+A_{A}\lambda_{A0}A_{A}\lambda_{A0}\right]+T_{B}\left(0\right)\left[A_{A}\lambda_{A0}A_{0}\lambda_{B0}\right]\\ T_{B}\left(2\right) & =T_{0}\left(0\right)\left[2A_{B}\lambda_{B0}-A_{B}\lambda_{B0}A_{0}\lambda_{A0}-A_{B}\lambda_{B0}A_{0}\lambda_{B0}-A_{B}\lambda_{B0}A_{B}\lambda_{B0}\right]\\ \; & \;+T_{A}\left(0\right)\left[A_{B}\lambda_{B0}A_{0}\lambda_{A0}\right]+T_{B}\left(0\right)\left[1-2A_{B}\lambda_{B0}+A_{B}\lambda_{B0}A_{0}\lambda_{B0}+A_{B}\lambda_{B0}A_{B}\lambda_{B0}\right]\\ T_{0}\left(2\right) & =T_{0}\left(0\right)\left[1-2A_{0}\lambda_{A0}-2A_{0}\lambda_{B0}+2A_{0}^{2}\lambda_{A0}\lambda_{B0}+A_{0}A_{A}\lambda_{A0}^{2}+A_{0}A_{B}\lambda_{B0}^{2}+A_{0}^{2}\lambda_{A0}^{2}+A_{0}^{2}\lambda_{B0}^{2}\right]\\ \; & \;+T_{A}\left(0\right)\left[2A_{0}\lambda_{A0}-A_{0}A_{A}\lambda_{A0}^{2}-A_{0}^{2}\lambda_{B0}\lambda_{A0}-A_{0}^{2}\lambda_{A0}^{2}\right]\\ \; & \;+T_{B}\left(0\right)\left[2A_{0}\lambda_{B0}-A_{0}A_{B}\lambda_{B0}^{2}-A_{0}^{2}\lambda_{A0}\lambda_{B0}-A_{0}^{2}\lambda_{B0}^{2}\right] \end{array}$$

The system of equations corresponding to the time instant *t* = 2 is Equation (15), in which the temperature values belonging to the time instant *t* = 1 appear. From the combination of the systems of Equations (14) and (15), the system of Equation (16) was obtained. (The physical meaning of the symbolic expressions of the unknowns appearing in the equations of systems (14)–(16) has already been defined earlier. See earlier.)

The slopes of the functions of the temperature variations are given by the differences of systems of Equations (13), (14), and (16) as follows: the difference of the last terms of the systems of Equations (13) and (14), respectively, the difference of the last terms of the systems of Equations (14) and (16), gives the slope of the first two sections of the broken-line type function curve of the temperature variation in the central material element, in the above order. Similarly, the slopes for the other material elements are determined accordingly. Since the temperature of the central element appears in each of the heat conduction equations, it is sufficient to examine only the heat conduction equation written for the central material element.

The slope of the first section of the mentioned temperature variation function curve is $\;T_{0}$(1) −$\;T_{0}$(0); the slope of the second section is $T_{0}$(2) −$\;T_{0}$(1). The slope of the first section is greater than that of the second section, since the amount of heat transported decreases over time during the process. According to the construction, it is true that if the temperature of the central element increases during the temperature variation, then the slope will always have a positive value, while if the temperature certainly decreases, then the slope will be negative. In a stationary thermal state, the difference is 0.

Based on this, if the ratio of the slopes thus calculated is formed, then the function of temperature variation can be characterized by the following four cases:

(a) If it can be written for the slopes that
  (17) $$\frac{T_{0}\left(2\right)-T_{0}\left(1\right)}{T_{0}\left(1\right)-T_{0}\left(0\right)}\geq 1$$
  then the values of the terms of the sequence composed of the slopes corresponding to the individual sections of the broken-line type function increase in time. In this case, the function is considered or called unstable, since in the closed thermodynamic system the speed of the heat conduction transport process shows a decelerating character in time. In this case, the function values diverge to positive infinity.
(b) If it can be written for the slopes that
  (18) $$1>\frac{T_{0}\left(2\right)-T_{0}\left(1\right)}{T_{0}\left(1\right)-T_{0}\left(0\right)}>0$$
  then the values of the terms of the sequence composed of the slopes corresponding to the individual sections of the broken-line type function decrease in time, but while retaining the decreasing character, they remain positive throughout. In this case, the function is considered and called stable, because in the closed thermodynamic system the stationary thermal state develops among the material elements.
(c) If it can be written for the slopes that
  (19) $$\frac{T_{0}\left(2\right)-T_{0}\left(1\right)}{T_{0}\left(1\right)-T_{0}\left(0\right)}=0$$
  then the broken-line type function reaches the stationary-state function value already at the first time instant (i.e., time step; *t* = 1). From the point of view of the model, this case is called the ideal case. This designation is justified because in this case this new—stationary—state can be determined with a single calculation.
(d) Finally, if it can be written for the slopes that
  (20) $$\frac{T_{0}\left(2\right)-T_{0}\left(1\right)}{T_{0}\left(1\right)-T_{0}\left(0\right)}<0$$

then the signs of the terms of the sequence composed of the slopes corresponding to the individual sections of the broken-line type function change in time. In this case as well, the function is considered and called unstable because in this closed thermodynamic system the nature of the temperature variation cannot be oscillatory monotonic (that is, the temperature cannot sometimes decrease and at other times increase).

To further aid the understanding of the four cases above, the following explanation is given: If the ratio of the slopes is greater than 1, that is, if the absolute value of the slope of the second section is greater than that of the first, then the temperature variation accelerates. In other words, in this case the temperature of the material element tends toward positive or negative infinity depending on whether the differences in the numerator and denominator of the fraction formed from the slopes are positive or negative in sign. The related limiting case is when the ratio is exactly 1. In this case the rise in the temperature does not accelerate, but its value tends toward positive or negative infinity.

If the value of the ratio formed from the slopes is less than 0, then the slopes of the examined sections of the temperature variation change sign. In the example shown in Figure 4, the denominator of the fraction is positive, since the temperature of the central material element increases compared to the zeroth time instant. However, the temperature will subsequently decrease, so the difference of the temperatures calculated for the second and first time instants (time steps) will be negative. Thus, in this case the settling will be oscillatory. In inequality (20) it is not indicated, but its limiting case is when the ratio becomes −1. In this limiting case, the temperature changes periodically with constant amplitude.

If the ratio of our fraction is less than −1, then the amplitude of the periodic variation oscillates between positive and negative infinity, changing constantly in time. If the value of the ratio formed from the slopes falls between 1 and 0, then the temperature reaches the stationary state according to a monotonic function with gradually decreasing slope, as shown in Figure 5. Its limiting case is the case when the value of the numerator of the fraction is 0. Then, naturally, the mathematical value of the entire ratio is also 0. This case occurs when $T_{0}\left(1\right)=T_{0}\left(2\right).$ In other words, when in the closed thermodynamic system there exists a locally stationary state (meaning a stable state from which, if the system is perturbed, it can only move toward a lower-energy but still stable state), in which case the change in the internal energy of the central material element per time instant (time step) will be zero.

By understanding the above, the next step in the optimization of the model is that among the above cases list in Section 3.2, we shall use case lists (c) in Section 3.2 in the following because it was established that from the point of view of model construction it is the ideal case. Thus, considering case list (c) in Section 3.2, into Equation (19) the values of the temperature of the central, i.e., zeroth, material element determined by the systems of Equations (13), (14), and (16) can be substituted. From the variables $\;B_{0}$, $\;B_{A}$, and $\;B_{B}$ appearing in these equations, the ratio of the time duration (Δt) and the square of the step length (Δx^2^), i.e., [Δt/Δx^2^], can be factored out. The −1st power of the product of mass density and heat capacity is denoted by $\;B_{0}$, $\;B_{A}$, $\;B_{B}$. After factoring out the multiplier [Δt/Δx^2^] and rearranging the equation, the following equality (21) results:

(21) $$\begin{array}{cc} \; & T_{0,n}\left(0\right)\;\left[2B_{0}^{2}\lambda_{A0}\lambda_{B0}+B_{0}B_{A}\lambda_{A0}^{2}+B_{0}B_{B}\lambda_{B0}^{2}+B_{0}^{2}\lambda_{A0}^{2}+B_{0}^{2}\lambda_{B0}^{2}\right]\cdot \frac{\Delta t}{\Delta x^{2}}\\ \; & \;+T_{A}\left(0\right)\;\left[-B_{0}B_{A}\lambda_{A0}^{2}-B_{0}^{2}\lambda_{B0}\lambda_{A0}-B_{0}^{2}\lambda_{A0}^{2}\right]\cdot \frac{\Delta t}{\Delta x^{2}}\\ \; & \;+T_{B}\left(0\right)\;\left[-B_{0}B_{B}\lambda_{B0}^{2}-B_{0}^{2}\lambda_{A0}\lambda_{B0}-B_{0}^{2}\lambda_{B0}^{2}\right]\cdot \frac{\Delta t}{\Delta x^{2}}\\ \; & =T_{0,n}\left(0\right)\;\left[B_{0}\lambda_{A0}+B_{0}\lambda_{B0}\right]+T_{A}\left(0\right)\;\left[-B_{0}\lambda_{A0}\right]+T_{B}\left(0\right)\;\left[-B_{0}\lambda_{B0}\right] \end{array}$$

By applying the Method of Equating Coefficients, from Equation (21) the system of inequalities (22), consisting of three identical inequalities, can be derived:

(22) $$\begin{array}{cc} \frac{\Delta t}{\Delta x^{2}}\; & \leq \;\frac{B_{0}\lambda_{A0}+B_{0}\lambda_{B0}}{2B_{0}^{2}\lambda_{A0}\lambda_{B0}+B_{0}B_{A}\lambda_{A0}^{2}+B_{0}B_{B}\lambda_{B0}^{2}+B_{0}^{2}\lambda_{A0}^{2}+B_{0}^{2}\lambda_{B0}^{2}}\\ \frac{\Delta t}{\Delta x^{2}}\; & \leq \frac{B_{0}\lambda_{A0}}{B_{0}B_{A}\lambda_{A0}^{2}+B_{0}^{2}\lambda_{B0}\lambda_{A0}+B_{0}^{2}\lambda_{A0}^{2}}\\ \frac{\Delta t}{\Delta x^{2}}\; & \leq \frac{B_{0}\lambda_{B0}}{B_{0}B_{B}\lambda_{B0}^{2}+B_{0}^{2}\lambda_{A0}\lambda_{B0}+B_{0}^{2}\lambda_{B0}^{2}} \end{array}$$

In the identical inequalities (22), on the left-hand sides stands the ratio of the parameters determining the run of the model [Δt/Δx^2^], and on the right-hand sides stand the physical parameters characterizing the material elements. All this therefore means that we are able to determine the optimal values of our model parameters with the help of the physical parameters characteristic of the material elements.

For the verification of the obtained result, the necessary physical parameters of silver, rock salt, and HDPE were substituted into the system of identical inequalities (22). While, similarly to the previous two examples, a Δx = 0.010 m differentiation distance was set, the system of equations yielded Δt = 13.871 s, 13.385 s, and 21.192 s. Checking the validity of these results, we obtained that the value given by the first identical inequality of (22) is the ideal one, since at the time instants (time steps) t(1) and t(2) the temperature of the central material element turns out to be nearly identical. The result (Δt) obtained from the second identical inequality is smaller compared to the value obtained in the first, i.e., ideal case. At the time instant (time step) t(2), the temperature is higher than the value obtained at the previous time instant t(1). With this described differentiation time setting, the operation of the model is sufficient (that is, not optimal, but corresponding to the real temperature variation function).

In the third case, the temporal variation in the temperatures gives a periodically changing, oscillatory character; therefore, this solution, according to the earlier justification list (d) in Section 3.2, is incorrect. The result of 18.751 s obtained for the ideal case falls between the differentiation times applied in the examples shown in Figure 4 and Figure 5.

**Figure 6:**
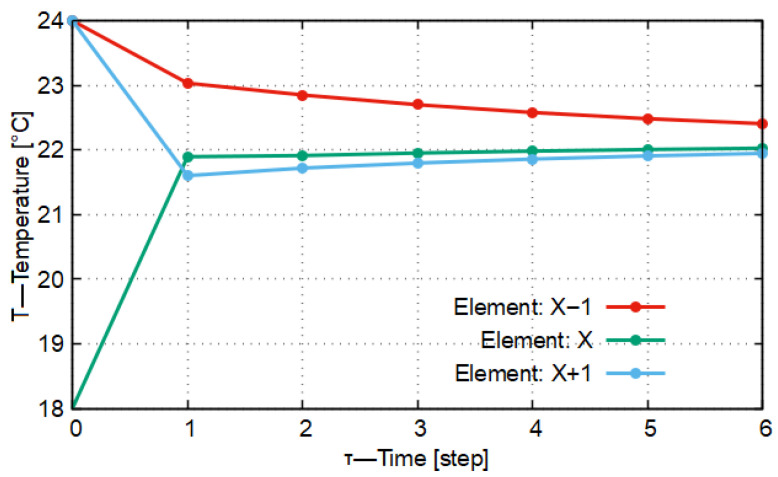
The behavior of a three-element (silver, rock salt, HDPE material) general-case 1D heat conduction model in the first time steps when the K is near the No-Sway Threshold. The temperature marked in green corresponds to the central element (rock salt), while the temperature marked in red (silver) and blue (HDPE) corresponds to its each neighboring elements.

By calculating the first five iterations of the ideal case and representing the results visually, the broken lines shown in Figure 6 are obtained, as the temperatures depend on the time steps.

In Figure 6 the optimal solution is shown, that is, the case in which, by applying a given differentiation distance (Δx), the stationary state can be determined with the least amount of computation, while the temperature variation does not have the previously detailed periodically oscillating character and closely follows the heat conduction process observable in nature.

In Figure 6 it can also be seen that the temperature change continues, since the heat capacity of the material element identified as X−1 (the result curve drawn with red line color in Figure 6) is greater than that of the other two material elements (i.e., the central and the other outer material elements). The target temperature, that is, the system temperature taken in the stationary state from the point of view of heat conduction, will be 22.222 °C, which the entire thermodynamic system reaches at the 64th time instant (time step), that is, after 887.750 s.

The system of identical inequalities (22) can be extended to two and three spatial dimensions. In both of these cases it is assumed that the differentiation distance is identical in every direction of the coordinate system, that is, Δx=Δy=Δz. Due to the extent of the derivations, we present here only the final result. We note that the derivation of this general case is identical to that already presented for the one-dimensional case using the systems of equalities and inequalities (13) and (22). The only difference is that in the case of the systems of Equations (13)–(16), they consist not of three but of seven equations, since in three dimensions the central material element is bordered not by two but by six neighboring material elements. From the results of the derivations for one-, two-, and three-dimensional cases, the following general dimension-independent formula can be deduced:

(23) $$\begin{array}{cc} \frac{\Delta t}{\Delta x^{2}}\; & \leq \frac{\sum_{i=1}^{2n}B_{0}\lambda_{i0}}{\sum_{i=1}^{2n}\sum_{j=1}^{2n}B_{0}^{2}\lambda_{i0}\lambda_{j0}+\sum_{i=1}^{2n}B_{0}B_{i}\lambda_{i0}^{2}}\\ \frac{\Delta t}{\Delta x^{2}}\; & \leq \frac{B_{0}\lambda_{i0}}{\sum_{j=1}^{2n}B_{0}^{2}\lambda_{i0}\lambda_{j0}+B_{0}B_{i}\lambda_{i0}^{2}} \end{array}$$

(In the (23) identical inequalities, *O* (origo) is the middle element, *n* is the dimension number, and *i* is the neighboring element.)

With the system of identical inequalities (23), the optimal or near-optimal differentiation time (Δt) and differentiation distance (Δx, since Δx=Δy=Δz) can be determined both for the one spatial dimension and even for the three general spatial dimension cases. It can be applied to every model where the size of the differentiation distance is identical for all material elements making up the model, and this condition is fulfilled for every spatial dimension (Δx=Δy=Δz).

In cases when the element sizes of the material elements constituting the heat conduction model are not identical per dimension (that is, Δx≠Δy≠Δz), then the system of identical inequalities (23) is no longer applicable. In the generalization of model construction, the use of material elements of non-identical sizes may also be necessary. In order to be able to interpret different magnitudes of element sizes within the model, we must return to the derivation of the two- and three-dimensional cases. In the general two- and three-dimensional cases, and in the general formula given in (23) derived from them, we always applied the assumption Δx=Δy=Δz.

In the earlier part of this publication, between (22) and (23), the complete derivation for one spatial dimension was presented. A similar derivation for the two- and three-dimensional cases was not included due to its length, but we note here that the course of that derivation is identical with the one applied in the already presented one-dimensional case.

The system of Equation (24) describes two-dimensional heat conduction, in which notations similar in meaning to those used in the previous examples are applied, taking into account that in two spatial dimensions the central material element (denoted with lower index 0) has four neighboring material elements (the number of rectangles touching the four sides of a rectangle is four), whose notations are in turn lower-indexed A,B,C,D.

The system of Equation (24) gives the temperatures $\;T_{\mathrm{An}}$, $\;T_{\mathrm{Bn}}$, $\;T_{\mathrm{Cn}}$, $\;T_{\mathrm{Dn}}$ of the four material elements neighboring a fixed central material element as functions of time in the *n*-th time step.

(24) $$\begin{array}{cc} T_{A,n} & =T_{A,n-1}+\frac{\Delta t}{\Delta x^{2}}B_{A}\lambda_{A0}\;\left(T_{O,n-1}-T_{A,n-1}\right)\\ T_{B,n} & =T_{B,n-1}+\frac{\Delta t}{\Delta x^{2}}B_{B}\lambda_{B0}\;\left(T_{O,n-1}-T_{B,n-1}\right)\\ T_{C,n} & =T_{C,n-1}+\frac{\Delta t}{\Delta y^{2}}B_{C}\lambda_{C0}\;\left(T_{O,n-1}-T_{C,n-1}\right)\\ T_{D,n} & =T_{D,n-1}+\frac{\Delta t}{\Delta y^{2}}B_{D}\lambda_{D0}\;\left(T_{O,n-1}-T_{D,n-1}\right)\\ T_{0,n} & =T_{0,n-1}+\frac{\Delta t}{\Delta x^{2}}B_{0}\left[\lambda_{A0}\;\left(T_{A,n-1}-T_{0,n-1}\right)+\lambda_{B0}\;\left(T_{B,n-1}-T_{0,n-1}\right)\right]\\ \; & \;+\frac{\Delta t}{\Delta y^{2}}B_{0}\left[\lambda_{C0}\;\left(T_{C,n-1}-T_{0,n-1}\right)+\lambda_{D0}\;\left(T_{D,n-1}-T_{0,n-1}\right)\right] \end{array}$$

The heat conduction model from this point on already assumes that the central material element’s lengths per dimension are not equal, that is, Δx≠Δy. We assume that there exists another system, similar to the above heat conduction system, which has physical parameters different from the heat conduction parameters appearing in the system of Equation (24), and in which the lengths per dimension of the material elements constituting the two-dimensional system are identical (that is, Δx=Δy), but the temporal change in heat conduction between the elements occurs in an identical manner and magnitude in both systems. In this case, we correspond the model to another model with a uniform lattice spacing as follows (see the system of Equation (25)):

(25) $$\begin{array}{cc} T_{A,n} & =T_{A,n-1}+\frac{\Delta t}{R}B_{A}\left[P_{AB}\;\lambda_{A0}\;\left(T_{O,n-1}-T_{A,n-1}\right)\right]\\ T_{B,n} & =T_{B,n-1}+\frac{\Delta t}{R}B_{B}\left[P_{AB}\;\lambda_{B0}\;\left(T_{O,n-1}-T_{B,n-1}\right)\right]\\ T_{C,n} & =T_{C,n-1}+\frac{\Delta t}{R}B_{C}\left[P_{CD}\;\lambda_{C0}\;\left(T_{O,n-1}-T_{C,n-1}\right)\right]\\ T_{D,n} & =T_{D,n-1}+\frac{\Delta t}{R}B_{D}\left[P_{CD}\;\lambda_{D0}\;\left(T_{O,n-1}-T_{D,n-1}\right)\right]\\ T_{0,n} & =T_{0,n-1}+\frac{\Delta t}{R}B_{0}[P_{AB}\;\lambda_{A0}\;\left(T_{A,n-1}-T_{0,n-1}\right)+P_{AB}\;\lambda_{B0}\;\left(T_{B,n-1}-T_{0,n-1}\right)\\ \; & \;\;\;\;+P_{CD}\;\lambda_{C0}\;\left(T_{C,n-1}-T_{0,n-1}\right)+P_{CD}\;\lambda_{D0}\;\left(T_{D,n-1}-T_{0,n-1}\right)] \end{array}$$

In this system, let the size of each element be $\Delta x=\Delta y=\sqrt{R}$, where $R\in R^{+}$. Then, the system of Equation (24) can be made equivalent to the system of Equation (25), in which the factor Δt/R appearing in front of the square bracket has been factored out. In this case, $R=\frac{\Delta x^{2}\;\Delta y^{2}}{\Delta x^{2}+\Delta y^{2}}$. We obtained this value by bringing the expressions $\Delta t/\Delta x^{2}$ and $\Delta t/\Delta y^{2}$, which stand before the terms inside the bracket, to a common denominator. After the factoring, inside the bracket each term includes, in accordance with the earlier multiplier, the proportionality factor $P_{AB}=\frac{\Delta x^{2}+\Delta y^{2}}{\Delta y^{2}}$, respectively, $P_{CD}=\frac{\Delta x^{2}+\Delta y^{2}}{\Delta x^{2}}$.

The system of Equation (25) assumes a system composed of cube-shaped material elements of identical edge length, starting from the system of Equation (24). Since from this point the steps of the derivation are identical to those applied for Equations (13) and (22) and the corresponding systems of equalities and inequalities, we do not provide the further derivation in full detail, but only present the final result. The steps of the derivation that are not explicitly written out were as follows: determination of the first two sections of the function of temperature change, examination of the monotonicity of these sections, description of the second section of the function of temperature change for zero slope, that is, $T_{0}\left(2\right)-T_{0}\left(1\right)=0$, and determination of the extreme values of the model parameters using the Method of Equating Coefficients in a form identical to that carried out for the system of equalities (22). At the end of the derivation, for two-dimensional heat conduction with material elements of variable edge length, the following system of equalities was obtained:

(26) $$\begin{array}{cc} \frac{\Delta t}{R}\; & \leq \frac{\sum_{i=1}^{2n}B_{0}\lambda_{i0}}{\sum_{i=1}^{2n}\sum_{j=1}^{2n}B_{0}^{2}P_{i}\lambda_{i0}P_{j}\lambda_{j0}+\sum_{i=1}^{2n}B_{0}B_{i}P_{i}\lambda_{i0}^{2}}\\ \frac{\Delta t}{R}\; & \leq \frac{B_{0}\lambda_{i0}}{\sum_{j=1}^{2n}B_{0}^{2}P_{i}\lambda_{i0}P_{j}\lambda_{j0}+B_{0}B_{i}P_{i}\lambda_{i0}^{2}}\\ P_{AB} & =\frac{\Delta x^{2}+\Delta y^{2}}{2\Delta y^{2}},\;P_{CD}=\frac{\Delta x^{2}+\Delta y^{2}}{2\Delta x^{2}},\;R=\frac{2\Delta x^{2}\Delta y^{2}}{\Delta x^{2}+\Delta y^{2}} \end{array}$$

(In the (26) identical inequalities, *O* (origo) is the middle element, *n* is the dimension number, and *i* is the neighboring element.)

The full derivation of the three-dimensional case is likewise not presented here due to its length. The methodology of the derivation is identical to that listed for the two-dimensional case and to that already presented for the one-dimensional, equal-edge-length case by the systems of equations and equalities–inequalities (13)–(22). As before, the model can then be brought into the general form given in (27) for the expression in three spatial dimensions. All the notations used here correspond in logic to the notation technique applied earlier.

(27) $$\begin{array}{cc} \frac{\Delta t}{R}\; & \leq \frac{\sum_{i=1}^{2n}B_{0}\lambda_{i0}}{\sum_{i=1}^{2n}\sum_{j=1}^{2n}B_{0}^{2}P_{i}\lambda_{i0}P_{j}\lambda_{j0}+\sum_{i=1}^{2n}B_{0}B_{i}P_{i}\lambda_{i0}^{2}}\\ \frac{\Delta t}{R}\; & \leq \frac{B_{0}\lambda_{i0}}{\sum_{j=1}^{2n}B_{0}^{2}P_{i}\lambda_{i0}P_{j}\lambda_{j0}+B_{0}B_{i}P_{i}\lambda_{i0}^{2}}\\ P_{AB} & =\frac{\Delta x^{2}\Delta y^{2}+\Delta x^{2}\Delta z^{2}+\Delta y^{2}\Delta z^{2}}{3\Delta y^{2}\Delta z^{2}},\;P_{CD}=\frac{\Delta x^{2}\Delta y^{2}+\Delta x^{2}\Delta z^{2}+\Delta y^{2}\Delta z^{2}}{3\Delta x^{2}\Delta z^{2}},\\ P_{EF} & =\frac{\Delta x^{2}\Delta y^{2}+\Delta x^{2}\Delta z^{2}+\Delta y^{2}\Delta z^{2}}{3\Delta x^{2}\Delta y^{2}},\;R=\frac{3\Delta x^{2}\Delta y^{2}\Delta z^{2}}{\Delta x^{2}\Delta y^{2}+\Delta x^{2}\Delta z^{2}+\Delta y^{2}\Delta z^{2}} \end{array}$$

(In the (27) identical inequalities, *O* (origo) is the middle element, *n* is the dimension number, and *i* is the neighboring element.)

### 3.3. General Numerical Examples—One-Dimensional Cases

The systems of identical inequalities (22) and (23) can be used for the determination of the optimal values of the model parameters in general cases. In the event of a heat conduction case involving three material elements of different physical properties, the No-Sway Threshold defined by the system of identical inequalities (9) is sufficient, but it does not return the most optimal discretization parameters (Δt and Δx). In the first two time instances (time steps), the temperatures of the material elements are calculated. Thereafter, with the slopes determined from these values, it can already be proven (the temperatures calculated at the first and second time instances will coincide) that the formula defined for the general case by (22) and (23) is also applicable for homogeneous material systems.

As an application, we tested the model with 22 different combinations of material elements. Taking into account the total number of possible combinations, we can state that we examined 10,648 cases. For each such case, our software determined the target temperature to a precision of three decimal places. From the results of these runs, we present a few examples here.

**Figure 7:**
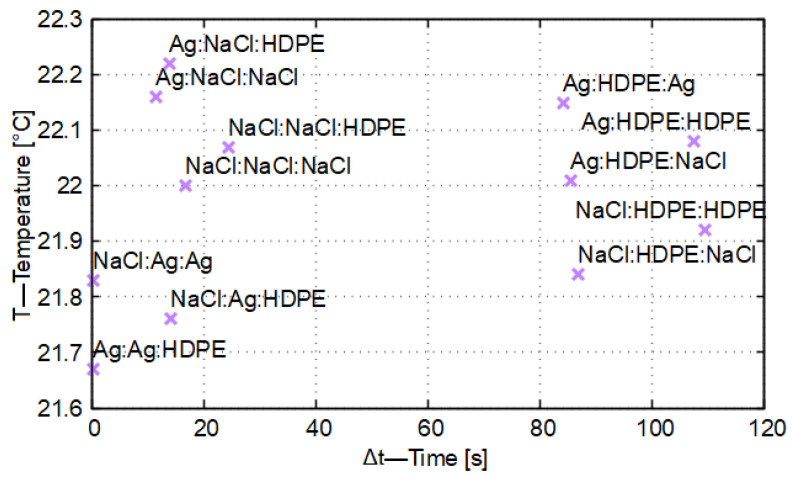
Some examples of the optimal differentiation time and differentiation distance for different material combinations in a three-element general-case 1D heat conduction model when *K* is near the No-Sway Threshold.

In Figure 7, the discretization time (Δt) is plotted as a function of the target temperature (*T*) for the pairwise combinations of silver, rock salt, and HDPE. The discretization distance was set to Δx = 0.010 m in each examined case. In all cases, the first identical inequality of the system of identical inequalities provided the ideal value of the discretization time.

Repeating this investigation for the two-dimensional case and also for the three-dimensional case, it can be observed that not in every case does the solution of the first identical inequality provide the ideal discretization values. From this it may follow that it is not sufficient in every case to solve only the first identical inequality of the system of identical inequalities (23), but the solutions of the remaining identical inequalities are also necessary. If it is necessary to calculate the second identical inequality of the system of identical inequalities (23) as well, then in order to determine the ideal discretization parameters, these must be verified with the help of the calculation of the first two time instances (time steps).

This is performed by also calculating the temperature of the central material element for the first two time instances (time steps). Thus, we will have three temperature values. These three temperature value points determine two straight-line segments, each of which has a slope that can be calculated. For the correct solution, it is required that the signs of the slopes thus calculated be identical. If these signs are not identical, then the identical inequality yields an incorrect solution. The ideal setting is the one where the two temperatures are identical or nearly identical in value.

### 3.4. Grid Optimization

**Figure 8:**
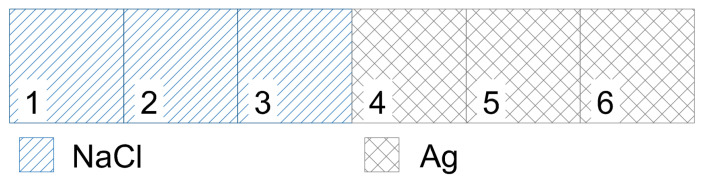
One-dimensional heat conduction model arrangement with two different materials, NaCl (blue elements) and Ag (silver elements), with their neighboring touching surfaces between #3 and #4 material elements.

Using the data from the previous examples, let us examine as an example the heat conduction between silver and table salt, for now in one spatial dimension. Let a one-dimensional model be given for our case, in which there are six material elements, of which three elements are silver and three are rock salt. The material elements in the model are arranged relative to each other as shown in Figure 8, that is, three rock salt elements are next to each other (the blue hatched areas in the figure) and three silver material elements (the black cross-hatched areas in Figure 8) also next to each other, and the contact between the two different types of material occurs between the third and fourth element pieces, thus modeling the contact boundary surface of the different types of materials.

Let the temperature of material elements 1–3 (NaCl) be *H*, and that of material elements 4–6 (Ag) be *L*. In the case of a model with uniform grid division, that is, when the discretization distance (Δx) is the same everywhere, the discretization time (Δt) can be determined based on the system of equal inequalities (21). Substituting into the (21) system of equal inequalities the given parameters of the two materials and the discretization distance, which in this example we take as Δx = 1 mm, the following discretization times are obtained as solutions, in order, for the six consecutive material elements: Δt = 16.78 s, 16.78 s, 11.54 s, 0.29 s, 0.19 s, and 0.19 s. Since in our heat conduction model different discretization times cannot be defined for a series of material elements participating in the heat conduction, the (21) equal inequalities are satisfied only if, among the different discretization times, the smallest value is taken as valid—in our case this is 0.19 s. We note that since this value was obtained for the fifth and sixth material elements, that is, for those having only silver material neighbors, the discretization time could also have been determined based on the first inequality of the system of equal inequalities (9), i.e., by observing the No-Sway Threshold.

**Figure 9:**
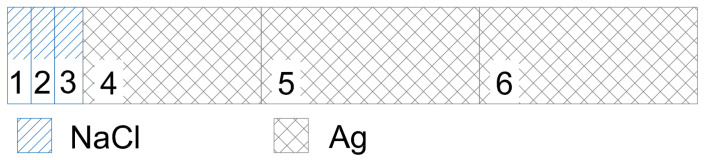
One-dimensional heat conduction model of two different materials in the case of the optimized mode, in other words, with optimized grid size between two different elements: NaCl (blue elements) and Ag (silver elements).

Now, let us examine the same case, but with the discretization time given initially (known) as Δt = 1 s. In this case, with the (21) equal inequalities, after substituting the parameters, the optimal discretization distances can be determined for the individual material elements. Performing the calculations, the following values are obtained for the discretization distances of the individual material elements: 2.44 mm, 2.44 mm, 2.94 mm, 18.4 mm, 22.6 mm, and 22.6 mm. If the model’s grid division is uniform everywhere (which initially was not assumed here), then the discretization distance of the model must be 22.6 mm according to the (21) equal inequalities. (In this case, from the series of obtained results, the largest value will be the appropriate one, since with a larger denominator, the value of the ratios is smaller.) In this case, i.e., if we were to choose a uniform grid division, the solution would remain suboptimal. In the case of the above example, the optimal heat conduction model can be achieved if the calculated discretization distance is applied as the size of the individual elements of the grid. If in the example model we do not change the number of material elements, then compared to Figure 8, the structure of the model changes as follows:

In Figure 9, the material elements are shown in linearly scaled distances, taking into account the discretization distances. The figure shows that the linear dimensions of the NaCl material elements are smaller, while the linear dimensions of the Ag material elements are larger. The linear length of the third material element in the sequence does not coincide with that of the first and second material elements, and likewise, the linear length of the fourth material element does not coincide with the linear dimensions of the fifth and sixth material elements. Where the model is homogeneous in material, the discretization distances—and thus the sizes of the elements—are identical. Differences arise only at the boundary surfaces. It can be said about the heat conduction model thus constructed with variable grid division (but constant in time) that it is optimized based on its parameters; that is, at certain points of the model, the temperature change function reaches the stationary state with fewer calculations, while the shape of the temperature change function develops according to real heat conduction. Using the previously introduced expression, the calculated temperature change follows a natural function.

The above solution, as also appears in the (21) system of identical inequalities, can be applied to two- or three-dimensional space. Let us examine such a multidimensional but real dissipative example. The next example is a proof-of-concept, which primarily compares the constant grid spacing solution resulting from our earlier derivations with the variable grid spacing solution that we have defined as ideal. Our goal with this is primarily to highlight the difference between the two models with different grid spacing, and it is not our aim to provide an exact solution to the posed engineering problem. For an exact engineering solution, in addition to heat conduction, flow and thermal radiation phenomena would also have to be modeled, which lies beyond the scope of the present paper. The comparison of the grid configurations is still a preliminary result and does not contain accuracy metrics relating to the results determined by the models. We plan to carry out the investigation of model accuracy in a later study, which according to our plans would already compare the simulated results derived from the mathematical model with real measurement data. In the present example—as will become clear from its description—we examine the model of a designed but not realized system, which therefore does not even provide an opportunity to perform comparative measurements.

The Flexblue® French-designed Small Modular Reactor (SMR) was planned as a cylindrical body, more precisely: 150 m in length and 14 m in diameter [35,51]. The project was to be realized through the joint cooperation of several organizations and companies, but following the 2008 concept, the project was nevertheless halted in 2016. The reasons for this included, among others, that based on the experience gained from the Fukushima Daiichi nuclear power plant disaster, the International Atomic Energy Agency (IAEA) tightened its regulations. An SMR placed beneath the sea surface carries a higher risk of terrorist attack. In the event of a malfunction, the isolation of radioisotopes and the decommissioning of the facility would be complicated by ocean currents. Moreover, during reactor operation, the waste heat released would considerably warm the surrounding water, and with this warming, the marine biome in the vicinity of the nuclear unit would change.

For the heat conduction analysis of Flexblue®, let us simplify the example itself. As a result of the simplification, suppose that we examine only the heat conduction between seawater and a steel pipe, while disregarding convection and other flows in the water. Since the detailed designs of the equipment are not public and thus not known, we also stimulate as assumptions of our model that we disregard the internal details and structural composition, and within the tank of 20 cm wall thickness we assume only air.

**Figure 10:**
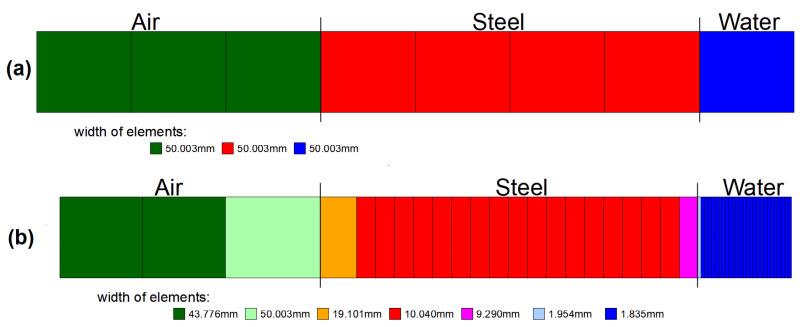
Flexblue external tank’s steel wall, single-line model: (**a**) fixed-grid-size model, (**b**) variable-grid-size model.

To compute the heat conduction of the outer wall of Flexblue®, we constructed both the uniform-grid model and the variable-grid model detailed previously. Figure 10 shows a single-line depiction representing one spatial-dimension direction for a 50.003 mm × 50.003 mm surface patch of the tank for these two models. The value of the 50.003 mm linear dimension resulted for the air material element at the air–steel media interface based on the solution of the (22) identical inequalities. In the above-mentioned uniform-grid model, every material element has dimensions 50.003 mm × 50.003 mm × 50.003 mm along the three principal spatial directions. In the variable-grid model, the surface size is 50.003 mm × 50.003 mm, but then the thickness of the material elements was determined with the equation expressing the R of the (27) identical inequality system, based on the optimal size for the given material element. Here, the optimal size was determined with the (22) identical inequalities.

In the spatial Cartesian (Descartes) frame of reference, the (22) identical inequality system can determine a differentiation distance only for a material element with equal edge lengths—i.e., cubes. We created material elements whose size differs in one spatial dimension by using the equation containing R from the (27) identical inequalities to scale the Δx × Δx × Δx element to Δξ × 50.003 mm × 50.003 mm, where ξ denotes the unknown thickness of the material element. By repeating this procedure for every material element, we obtain element b) of Figure 10.

**Figure 11:**
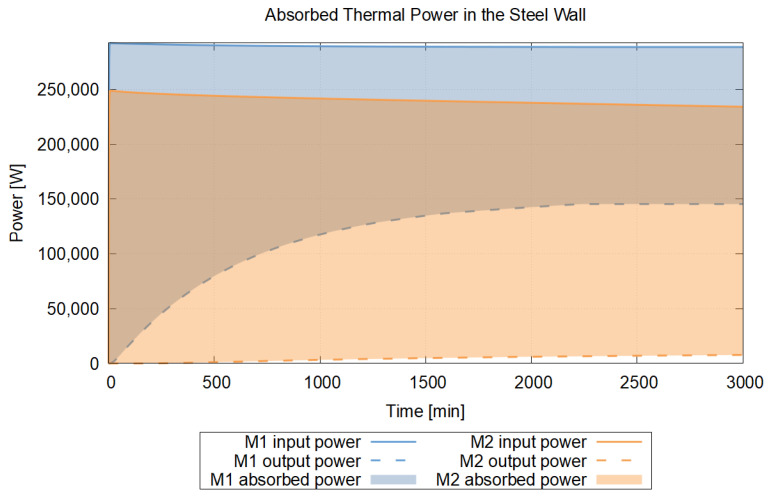
Heat absorbed in the Flexblue’s steel wall in the case of fixed-grid-size model (M1) and variable-grid-size model (M2).

In both heat conduction models used, we examined cases with the same initial conditions and parameters. The case is an emergency shutdown in the Flexblue® SMR, as a result of which the air temperature inside the tank instantly became 200 °C. An additional initial condition was that the temperatures of the water and the steel were both 24 °C, and the leftmost and rightmost material elements of the single-line model expressing the linear dimension shown in Figure 10 remained at constant temperature, that is, the temperature of the leftmost air material element was constantly 200 °C and the temperature of the rightmost seawater material element was constantly 24 °C for the entire simulated duration. The computational results, scaled up to the full surface, are shown in Figure 11.

As was mentioned above, only renderings of Flexblue® are available to the authors; therefore, in determining the total external surface, we assumed that the two ends of the pressure vessel with cylindrical spatial geometry are not hemispheres, but would have been made with two end terminations having half-ellipsoid surfaces. Taking all this into account in the calculations, we determined, for the total surface, a surface-area value of 6714.39 m^2^.

In Figure 11, we denoted the uniform-grid model by M1 (blue-colored domain), while the variable-grid model was denoted by M2 (orange-colored domain). The domains are bounded from above by the power–time curves of power taken up from the air and from below by the power–time curves of power delivered to the seawater. In Figure 11 it can be seen that, in the two cases, the outer wall of Flexblue® stores the heat power to different extents. In the case of the uniform-grid model, comparing the solid blue curve with the solid orange curve of the variable-grid model shows that, according to the M1 model, the wall of the tank takes up more heat power from the air than is seen in the result obtained by the M2 model. If we compare the blue and orange dashed-line curves, it can be seen that, in the case of the uniform-grid model (M1), the function of heat power delivered to the seawater increases faster than the similar function obtained in the variable-grid model (M2). These results show that, in the case of the M2 model, the taken-up heat power heats the metal to a greater extent than in the M1 model.

**Figure 12:**
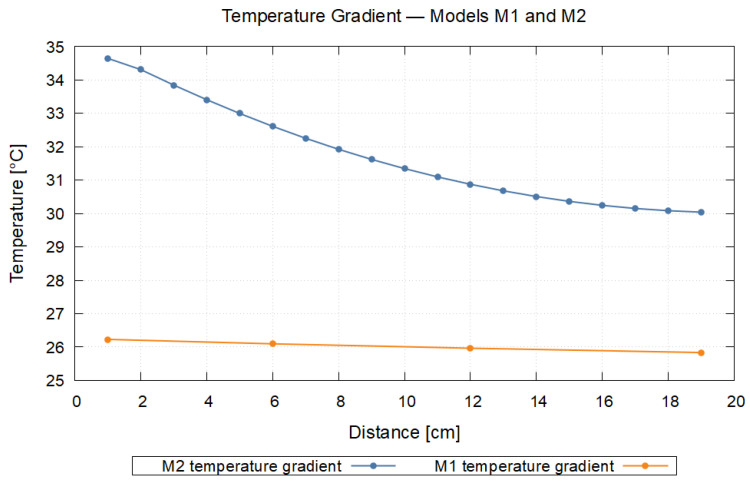
Temperature gradient through Flexblue’s steel wall in the case of fixed-grid-size model (M1) and variable-grid-size model (M2) at t = 3000min (the orange-colored curve contains only 4 data points, while the blue-colored curve contains 19 data points, where one data point highlights the temperature of a material element. Since in the M1 model the steel tank consists of 4 material elements, this is analogous to the appearance of 4 data points, whereas in the M2 model we can speak of 19 material elements, which means exactly 19 data points).

The difference between the heat-power uptake of the steel tank modeled in the two models is also demonstrated by the two graphs shown in Figure 12, which plot the temperature gradient at the 3000th min as a function of linear distance within the tank wall. Taking into account that the initial temperature of the steel tank was 24 °C, in the case of the uniform-grid model (M1) the temperature of the steel develops only between 26.229 °C and 25.835 °C. By contrast, the temperature gradient in the case of the variable-grid model (M2) varies between 34.648 °C and 30.041 °C within the wall. On this basis, in the case of the variable-grid model (M2), over the examined time domain a significant portion of the heat power was expended on the heating of the tank wall.

As a closing remark to the chapter, we consider it important to note that in this chapter we presented preliminary results that were produced by a sequence of calculations and are not yet simulation results run with our own, fully finished CHeTMoS target software. The sequence of calculations means using and computing, one after another, the equations and identical inequalities presented above—that is, precisely this series of recursive computations. The current version of our CHeTMoS software operates with uniform grid spacing. The possibility of applying variable grid spacing will be incorporated into our self-developed software following the present publication.

## 4. Summary

The study addresses the non-stationary cases of heat conduction modeling based on the explicit Euler method, starting from the classical Courant–Friedrichs–Lewy (CFL) stability condition and its forms referring to the heat conduction (Fourier) number, *K*. The paper shows that although the CFL is a necessary condition for computational stability, it may also allow oscillating (swaying) temperature–time curves in intermediate states, which differs from the natural transient behavior observed in reality with monotonic approximation.

Therefore, we introduced the concept of the No-Sway Threshold: a K’s values stricter than the CFL, which in every dimension prescribes a denominator greater by one compared to the denominator of the CFL (thus, in 1D $K\leq \frac{1}{3}$, in 2D $K\leq \frac{1}{5}$, and in 3D $K\leq \frac{1}{7}$). The No-Sway Threshold ensures that transient temperature distributions converge to the stationary state in a non-oscillatory, monotonic manner. The constancy of the energy content of the closed thermodynamic system was proven by a geometric argument: the signed sum of the per-time-step “triangle areas” defined according to (A1a) and (A1b) is zero.

The paper provides a methodology for determining the optimal discretization (Δt, Δx). The (22) system of identical inequalities written with the Method of Equating Coefficients computes the expedient time and space steps from the physical parameters of the medium ($\alpha =\frac{\lambda }{\rho c_{p}}$); this is applicable in homogeneous and inhomogeneous systems, as well as in multidimensional extensions ((23) in the case Δx = Δy = Δz).

The numerical examples presented confirm the theory. For homogeneous silver material elements, with Δt = 1 s, at Δx = 0.024 m, *K* = 0.336 (above the No-Sway Threshold) causes 0.028 °C overshoot relative to the target temperature, while at Δx = 0.0241 m, *K* = 0.333 (below the No-Sway Threshold), the transient is monotonic and overshoot-free. In the inhomogeneous example (Ag/NaCl/HDPE) the Δt that proves optimal around the No-Sway falls between 8.5 and 9.15 s; for the middle element, depending on the neighbor, *K* = 0.119 and 0.413 result, which supports the necessity of a differentiated treatment of parameter selection (based on (13)–(16) the slope analysis of the initial sections decides the monotonicity).

The presentation of grid optimization on a six-element (NaCl/Ag) 1D example shows that the smallest of the element-dependent Δt values calculated on the basis of (21) determines the uniform time step; in the reversed problem, for a given Δt element-dependent optimal Δx values result. The variable grid spacing formed thus yields smaller linear element sizes in the NaCl zone and larger in the Ag zone. Then, the model run is faster and involves a more natural temperature transient.

Finally, the manuscript notes that the CHeTMoS software currently handles uniform-grid models; the incorporation of support for variable grids is planned on the basis of the results.

## Figures and Tables

**Table 1:** Physical properties of the materials examined in the examples.

Material	Silver (Ag)	Rock Salt (NaCl)	HDPE	Seawater (20 °C and 35 g/kg)	Steel (100 °C)	Air (100 °C)
Thermal conductivity	419 W/mK	3.87 W/mK	0.45 W/mK	0.59 W/mK	16.14 W/mK	0.03095 W/mK
Specific heat capacity	234 J/kgK	900 J/kgK	2300 J/kgK	3998.9 J/kgK	504.8 J/kgK	1009 J/kgK
Mass density	10,500 kg/m^3^	2165 kg/m^3^	950 kg/m^3^	1024.9 kg/m^3^	8000 kg/m^3^	0.9458 kg/m^3^

Properties came from the following: silver [45]; rock salt [46]; HDPE [47]; seawater [48]; steel [49]; air [50].

**Figure A1:**
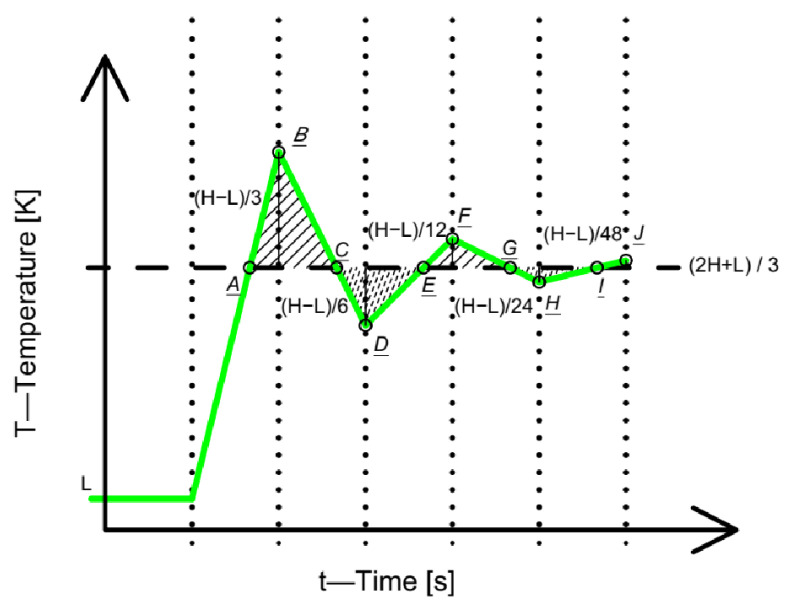
Temperature variation in the one-dimensional thermodynamic system containing three material elements during heat conduction as a function of time after five time-iteration steps. It shows the temperature of the middle element temperature changes in time.

**Figure A2:**
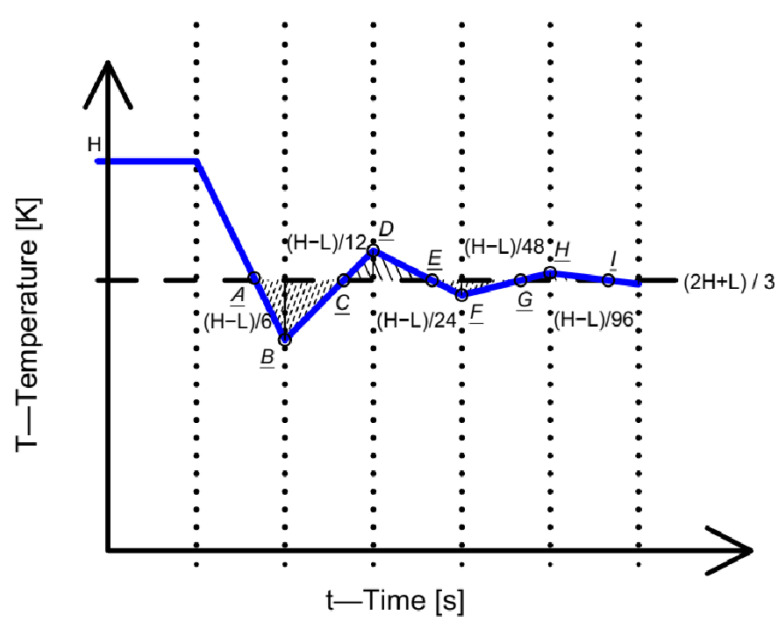
Temperature variation in the one-dimensional thermodynamic system containing three material elements during heat conduction as a function of time after five time-iteration steps. It shows the temperature of the side element(s) temperature changes in time.

## Data Availability

The data presented in this study are available on request from the corresponding author.

## Abbreviations

The following abbreviations are used in this manuscript:

CFL	Courant–Friedrichs–Lewy
CHeTMoS	Complex Heat Transfer Modeling Software
FDM	Finite Differences Method
FEM	Finite Element Method
FVM	Finite Volume Method
HDPE	High-Density Polyethylene
IAEA	International Atomic Energy Agency
SMR	Small Modular Reactor

## Appendix A. Constancy of System Energy

From Figure 1 and from the fact that the above target temperature is the arithmetic mean of the temperatures of the three adjacent material elements, it can be concluded that the internal energy of the system is constant. This can be proven by examining the amplitudes of the oscillations as follows:

In Figure 1, every value in the Δt time interval is known, that is, the vertices of the colored broken lines in the figure are known as temperature values. To prove the constancy of internal energy, we will define triangles in Figure 1. One such triangle is shown in Figure A1 as the triangle with vertices A, B, C, but similar ones are the triangles CDE, EFG, GHI, etc. The heights of these triangles are the perpendicular line segments drawn from the vertices of the broken line to the horizontal dashed line representing the target value (see the vertical continuous line segments in Figure A1). The side of the mentioned triangles corresponding to the marked heights has the length Δt, which, as we know, is the length of the discretization time interval. From the data described in this way, the areas of the triangles can then be determined.

As can also be seen, the graphs of the broken lines marked in green and blue describe the temperature variation, which is directly proportional to the change in internal energy. Let the constant expressing the direct proportionality be denoted by *P*. All this means that the change in internal energy in the system is described by a graph of similar geometry to that of the graph describing the temperature variation. Thus, if we were to examine the graph of the internal energy change, then the vertices of this broken line curve would provide the numerical values of the thermal work performed during the temperature variation up to the given $\Delta t_{n}$ time interval. Since the entire system tends toward the stable state in which all three adjacent material elements have the same temperature, the last local extremum will be the arithmetic mean of the internal energies of the three mentioned material elements. Taking the above into account, namely that the graph of temperature variation is directly proportional to the graph of internal energy variation and its proportionality factor is *P*, the final value of the internal energy after temperature equalization will be equal to the numerical value determined by Equation (8) multiplied by the proportionality factor *P*.

It can be stated that, as intersection points of the horizontal straight line parallel to the x-axis passing through the last extremum point, further points are created on the broken lines, the corresponding thermal work of which will be identical. Since the system is thermally closed, the algebraic sum of the thermal works performed on the three adjacent material elements between two such intersection points must be zero. (It is important to note that this will be true if no chemical-type work occurs and no other or any energy source is present in the system.) In Figure A1 and Figure A2, the algebraic sum of the areas of the triangles interpreted and introduced above—that is, physically, the thermal power corresponding to them—must be zero both separately over the individual Δt intervals and over the entire n·Δt intervals. The sign of the algebraic areas in this case can be interpreted as whether the given triangle is located below (negative area) or above (positive area) the horizontal dashed reference line.

Let us now deal in more detail with the determination of the areas of the triangles introduced above. As we have seen, in Figure A1 and Figure A2 the heights of the triangles are indicated. The side lengths corresponding to these heights are Δt in the case of each triangle; therefore, when summing the areas, these factors can in every case be factored out from the products expressing the areas. Equations (A1a) and (A1b) already express the thermal work ($W_{T}$) after the simplifications. Equation (A1a) gives the thermal work corresponding to the central material element, while Equation (A1b) gives the thermal work corresponding to the two boundary material elements. The factor of two at the beginning of expression (A1b) describes the number of the two boundary material elements.

We have shown above that the denominator of the final temperature value to be reached by the system is two (see Equation (8)), while the denominators of the local extrema of the temperature function are, respectively, three or even integer multiples thereof. It follows that the coefficients inside the square brackets in Equations (A1a) and (A1b) are, theoretically, infinite series. Since in the entire presented procedure only the first five terms were represented, in Equations (A1a) and (A1b) only the first five terms are included. From these it is also clearly visible that if we add expressions (A1a) and (A1b), then their algebraic sum will be zero.

(A1a) $$\begin{array}{cc} \frac{\Delta t}{2}\;\left[\frac{H-L}{3}-\frac{H-L}{6}+\frac{H-L}{12}-\frac{H-L}{24}+\frac{H-L}{48}\right] & =W_{T} \end{array}$$

(A1b) $$\begin{array}{cc} 2\cdot \frac{\Delta t}{2}\;\left[-\frac{H-L}{6}+\frac{H-L}{12}-\frac{H-L}{24}+\frac{H-L}{48}-\frac{H-L}{96}\right] & =W_{T} \end{array}$$
